# Potential seismic precursors and surficial dynamics of a deadly Himalayan disaster: an early warning approach

**DOI:** 10.1038/s41598-022-07491-y

**Published:** 2022-03-08

**Authors:** Anil Tiwari, Kalachand Sain, Amit Kumar, Jyoti Tiwari, Ajay Paul, Naresh Kumar, Chinmay Haldar, Sushil Kumar, Chhavi P Pandey

**Affiliations:** Wadia Institute of Himalayan Geology, 33, General Mahadev Singh Road, Dehradun, Uttarakhand 248001 India

**Keywords:** Natural hazards, Planetary science

## Abstract

On 7 February 2021, Chamoli district (Uttarakhand, India) was devastated by a deadly rock-ice avalanche that led to a large causality of more than 200 people and a huge economic loss. We found noteworthy sequence of precursory signals of main failure/detachment preceded by a dynamic nucleation phase. The rock-ice avalanche appears to have been initiated by seismic precursors which were continuously active for 2:30 h prior to main detachment. The seismic amplitude, frequency characteristics and signal-to-noise ratio variation of detected tremors indicate static to dynamic changes in nucleation phase located at the source of detached wedge. The characteristics of seismic data distinguished debris flow and hitting obstacles from other seismic sources and allowed the estimations of debris flow speed. We analyzed and verified the seismic signals with field evidences to estimate the associated impacts and velocity of dynamic flow. The proximal high-quality seismic data allowed us to reconstruct the complete chronological sequence and evaluate impacts since the initiation of nucleation phase to its advancement. Furthermore, we suggest that real-time seismic monitoring with existing network and future deployment of integrated dense network can be used for forecasting of flow events and hazard mitigation in the downstream.

## Introduction

The Himalayan glaciers are rapidly retreating due to increase in glacier melt and climate change in recent decades^1,2^. The retreating of glaciers and associated melt along with unstable slopes with steep topography are prone to landslide, which lead to development of lakes and subsequent flood events in the Himalaya^3,4^. Many of these developing lakes (dammed by ice-cored moraines) and unstable slopes are frequently subjected to trigger by intense monsoonal precipitation and potential seismic events in its close periphery. The Himalayan flash floods originating from the higher elevation (glacial terrain) pose a significant threat to humans in the downstream. The monitoring of Himalayan glaciers are challenging and expensive to access for scientific research purpose. The unfelt destructions produced by the glacier mass movement activities can only be recognized by satellite images, if other monitoring is not possible. The gradual process of surficial deformations i.e., mass movement in glacial terrain can accelerate with time to cause causality and economic loss in the downstream along the river. The movement can be recorded by highly-sensitive seismometers of a seismic network in different frequency-time domain. Moreover, the observation of these unfelt destructions are important to demarcate the potential endangered zone to avoid human settlement and other modern structures in the downstream. The recent catastrophic event of 2021 in Rishiganga–Dhauliganga valley caused a great causality of human and huge loss to economy. After this deadly event, a research organization, Wadia Institute of Himalayan Geology (WIHG) is more focused in monitoring all these significant and unfelt activities with a dense network of multi-parametric instruments like seismometers, automatic weather stations (AWS), infrasound array, automatic water level recorders (AWLR), etc., in the vicinity of Himalayan glaciers. A detailed study of these multi-parameters can achieve a strong correlation between exogenic and endogenic processes to understand the source dynamics of the region. Also, the observations of unfelt activities and surface–subsurface dynamicity recorded in the seismic network prior to main failure could achieve a probable mitigation response as an early warning system (EWS) in the region.

The seismic signals associated with active dynamic deformations i.e. earthquakes, volcanic eruptions, landslides, rock-falls, avalanches, etc. can be identified using the seismic waveform spectrum^5–10^. Some previous studies suggest that the seismic characterization of surficial dynamics for rock-ice avalanche and landslides is important for understanding the dynamicity and onset of the events^11,12^. These deformations generate ground motions with an efficient coupling into the ground and are recorded at the seismic stations. Depending on the location, the propagating seismic/acoustic signals can be observed for wide varieties of sources and recorded as seismic signals. The site effect also plays a prominent role in recording the seismic waveform which is controlled by the underlying ground or material property (topography, lithology, structure) and can affect the traverse even from near-source activities. It is observed that seismic energy generated in surficial activities is easily saturated or attenuated with distance, while seismic energy associated with subsurface ruptures can travel long distance^13,14^. Several studies have developed the relationship between high-frequency seismic signals, landslides, avalanches, debris flow, etc^15–19^. It has been identified that volume of destruction caused by dynamic activities can be linked to amplitude spikes or radiated energy in terms of high-frequency signals. The seismic wave amplitude is proportional to the force drop during the release of mass owing to kinetic energy and in the case of an earthquake, the seismic amplitude depends on seismic moment (force drop × source dimension). The snow avalanches and debris flow generate a systematic increase in high-frequency energy and are transmitted into the nearest seismic stations^10^. We acknowledged the recent scenario of new approaches and findings based on the investigation of high-frequency seismic signals^16,20–26^. These signals are generated independently of the seismic moment (size of the event) and can be recorded in the seismometers within the proximity of the source. Various experiments have been done which suggest the initiation of subsequent nucleation process leading to the material failure, during which rearrangement of deformed material can lead to creep or internal deformations such as cracks and fractures advancement^17,27–30^. The dynamicity of weak zone accelerates as time get close to main detachment or rupture. Sometimes, the potentially active zone generates continuous precursory signals long prior to the main event, which need to be identified with the source information (discussed in this study). The unfelt high frequency seismic energy released in glacier avalanche and debris flow travels to proximal stations with less attenuation and is restricted to distance within the periphery of source. Unfortunately, we are still facing a challenge to characterize the source with its accurate epicenter based on seismic waveform. The detailed prior information based on the identification of pre-signatures may lead to mitigation of economic loss and mass population.

**Figure 1:**
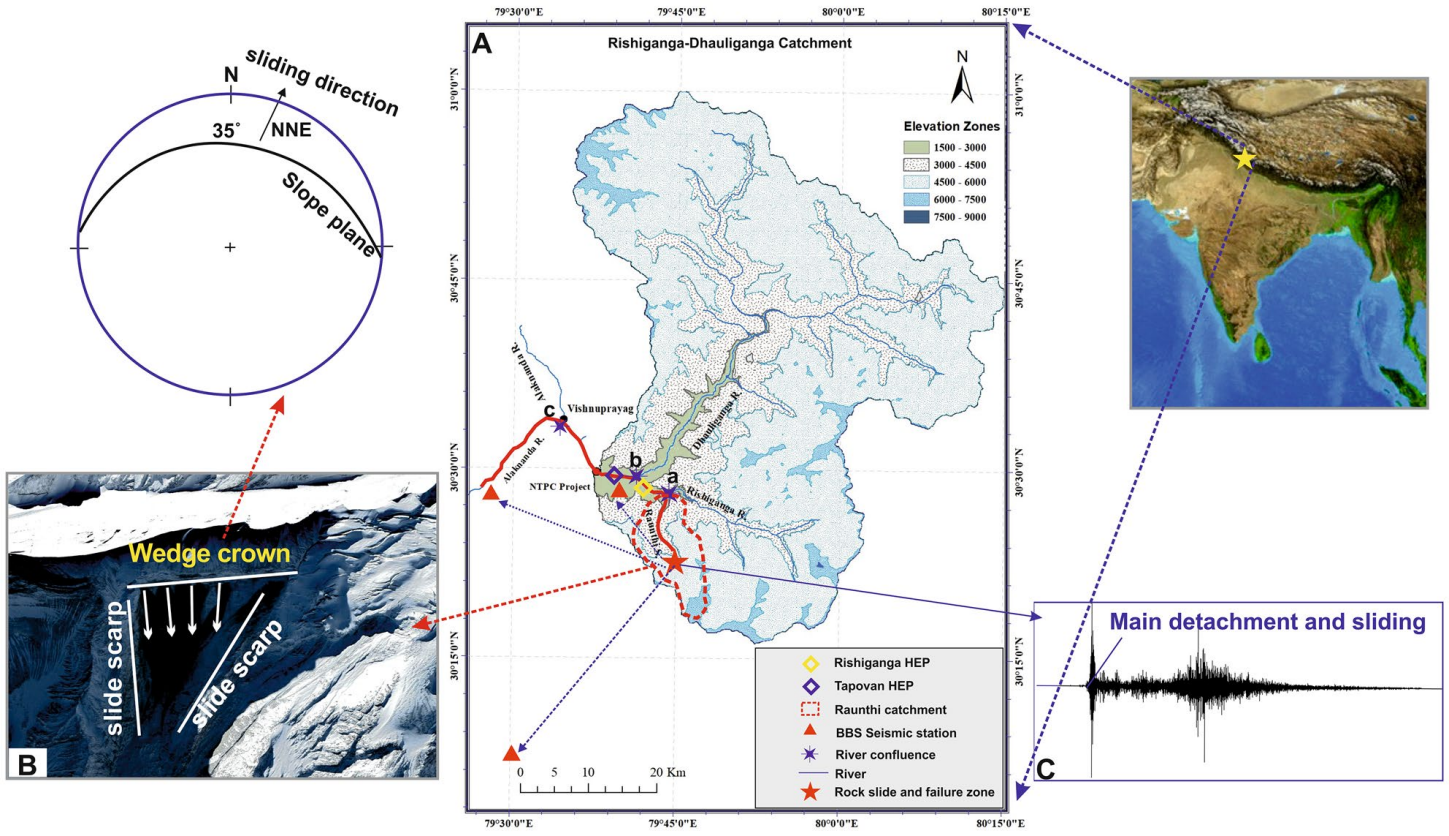
Rishiganga–Dhauliganga catchment. (**A**) Showing source/triggering zone of rock-ice avalanche inside the Raunthi catchment and subsequent affected path with modern structures along the river. (**B**) A satellite image showing source zone from where rock-ice material detached after failure and stereographic representation with the attributes of sliding plane. (**C**) Recorded waveform spectrogram in the nearest seismic station (12 km away from the main detachment), showing 1st phase of main failure at 4:51.10 UTC along with the noise of debris flow. Map was generated using ArcGIS software licensed version 10.5 (https://www.arcgis.com/index.html) and insert satellite image was downloaded from Google Earth Pro software version 7.3 (https://www.google.com/earth/versions).

On 7th February 2021, a deadly rock-ice avalanche led to a devastating flash flood in Raunthi Gad near Rishiganga-Dhauliganga valley in the Chamoli district of Uttarakhand state (Fig. 1 and Fig. S1). The weak detached material slides from 5525 m altitude in NNE aspect along the 35° slope of plane from the source zone. According to the source (www.indiatvnews.com), at least 72 people were found dead, 29 human body organs recovered and more than 200 people still missing. The impact of the flash flood was mainly constrained to the modern structures i.e. two hydropower projects, bridges and roads along the river path. The high-flow intensity of the flood disturbed the broad zone creating instability near the Raini village and the villagers have evacuated the village (Fig. S2). It is evident that the Raini village is unstable and requires rehabilitation of affected villagers. In June 2013, Uttarakhand has experienced another deadly Kedarnath event that coincided with peak tourist season during the pre-monsoon period. Almost 5860 people died (including missing bodies) in that drastic event^31^ (Source: Disaster Mitigation and Management Centre (DMMC), dated 17.09.2013). The maximum glacier-related hazards like avalanche, GLOF (glacial lake outburst flood), flash flood, debris flow, etc. took place during the peak ablation or summer season (June and July), while the present avalanche and flash flood occurred in the morning hours of peak winter season (February). Uttarakhand has a total of 1573 glaciers with total covering area of 2258 km^2^. The periphery of the recent avalanche comprises a total of 245 glaciers with glacier area of ~ 436 km^2^. Also, the area is in the vicinity of the major Himalayan thrust i.e. Main Central Thrust (MCT) and witnessed the 1999 Chamoli earthquake (Mb = 6.3)^32^. Uttarakhand state is seismically active (comes under seismic zone IV and V)^33^ and witnessed more than 15 major disasters from flash floods, earthquakes and landslides since the nineteenth century^34^.

The WIHG, Dehradun has been pursuing research to unravel the orogeny of the majestic Himalaya and provide an improved understanding on seismogenesis and geodynamics of the Himalaya. For that purpose, the Institute is continuously monitoring the seismicity using several broad-band seismic stations installed across the Himalaya. The WIHG is the only agency that has three highly sensitive broadband seismic stations since 2007 close to the avalanche release zone within 12–45 km distance (Fig. S1). The affected region are in the proximity of a major Himalayan thrust i.e. Main Central Thrust (MCT). The stations are close enough to detect any significant movement of glaciers and rocks in the Rishiganga–Dhauliganga valley. A noteworthy contribution to our current understanding of rock-ice avalanche has been made in Himalaya. The seismic stations record high-quality seismic data with a high signal-to-noise ratio which allows for a detailed reconstruction of deadly avalanche dynamics. After the recent deadly event,  numerous studies have been carried out,  which states the geometry of detachment, mechanism of rock-ice avalanche, flow velocity, downstream devastation, post impact of flash flood based on remote sensing and regional seismic network^35–38^. The present article investigates and highlights the complete scenario of pre and post dynamics of the source zone of material failure. The precursory seismic signals of the detachment with source location is extremely important to understand the degree of instability in the region. We present the seismic and associated field evidences for the 7th February 2021 Chamoli rock-ice avalanche. The study also finds seismic pre-signatures in the frequency–time–amplitude domain and subsequent ground coupling surface impacts for the natural dynamic events. This study reveals the accurate timing of rock-ice avalanche with different pre and post-stages of high frequency seismic tremors. We analysed the dynamicity of subsequent seismic tremors prior to the main event and provide improved understanding in assessing the weak zone static to dynamic nucleation process and associated impacts. The study also reports a verified chronological time-series sequence (relation between real-time seismic signatures with temporal dynamic activity connected to rock-ice avalanche and successive deformation due to huge mass debris flow) during and after avalanche release. Here we also suggest how the prior detection of seismic signals can provide a systematic approach and timely warnings to avoid the risks related to hazards.

## Avalanche release zone

A team of scientists from WIHG responded and moved to the Rishiganga–Dhauliganga valley (Fig. 1) on 8th February 2021 to make a reconnaissance of the event and understand the reasons behind the disaster. The source zone of avalanche and peripheral impacts were observed for the first time from a helicopter fly, immediately after the disaster. It was observed that a hanging glacier along with underlying rock mass broke off in the Raunthi Glacier valley below Raunthi Peak from an altitude of 5525 m asl and has a north–north-easterly (NNE) aspect. The rock-ice mass moved downward along a steep slope with relief of more than 2 km and hit Raunthi stream at an altitude of ~ 3500 m asl. The distance of this impact is about 1.6 km downstream from the snout of the Raunthi Glacier. The average inclination of the slope is nearly about 35° with a significant landscape relief of more than 2000 m. The calculated volume of ~ 27 × 10^6^ m^3^ slipped into a Rishiganga valley^35^ with high flow front velocity. The release zone comprises high-grade metamorphic rocks of Proterozoic age such as kyanitic schist, migmatite gneiss, and quartzite^39^. The temporal analysis of satellite images of the avalanche zone shows the gradual growth of cracks and joints near the crown of weak wedge that controlled the head scarp since the last 5 years^37^. The initiation and advancement of cracks and fractures had started to open after 2015 and indicates a successive expansion of weak zone near the crown of wedge failure (source zone). A steady process of fracture development within the periphery of steep slope had started years before the main activity of February 2021, which just needed to be activated by steady to active phase of dynamicity for huge material failure (discussed in present article).

## Precursory seismic tremors

**Figure 2:**
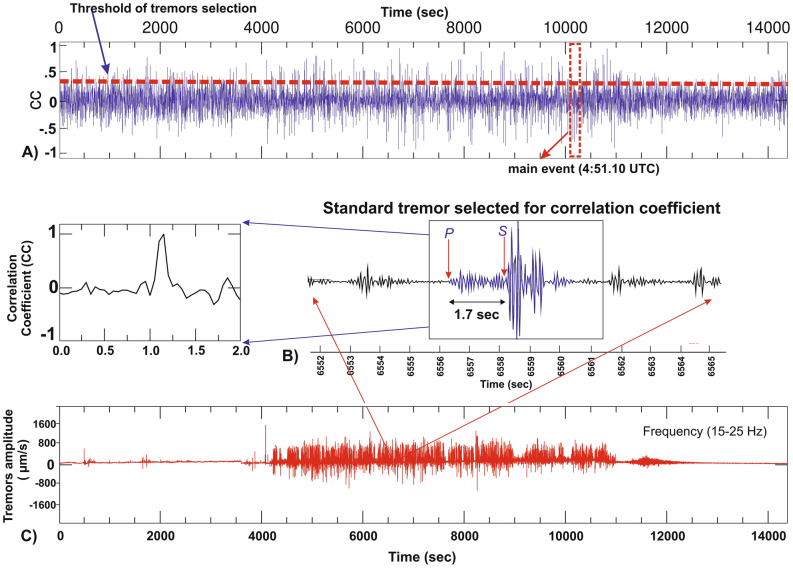
Correlation coefficient trace of precursory seismic signals. (**A**) The continuous sequence of precursory tremors follows the (red dashed line) threshold, above which precursory tremors are detected. (**B**) A standard tremor based on high signal-to-noise ratio (SNR) is selected for correlation coefficient trace in 4 h moving window. (**C**) The waveform spectrogram showing the dominancy of tremors amplitude after ~ 4000 s (~ after 3:00  UTC, 07/02/2021).

**Figure 3:**
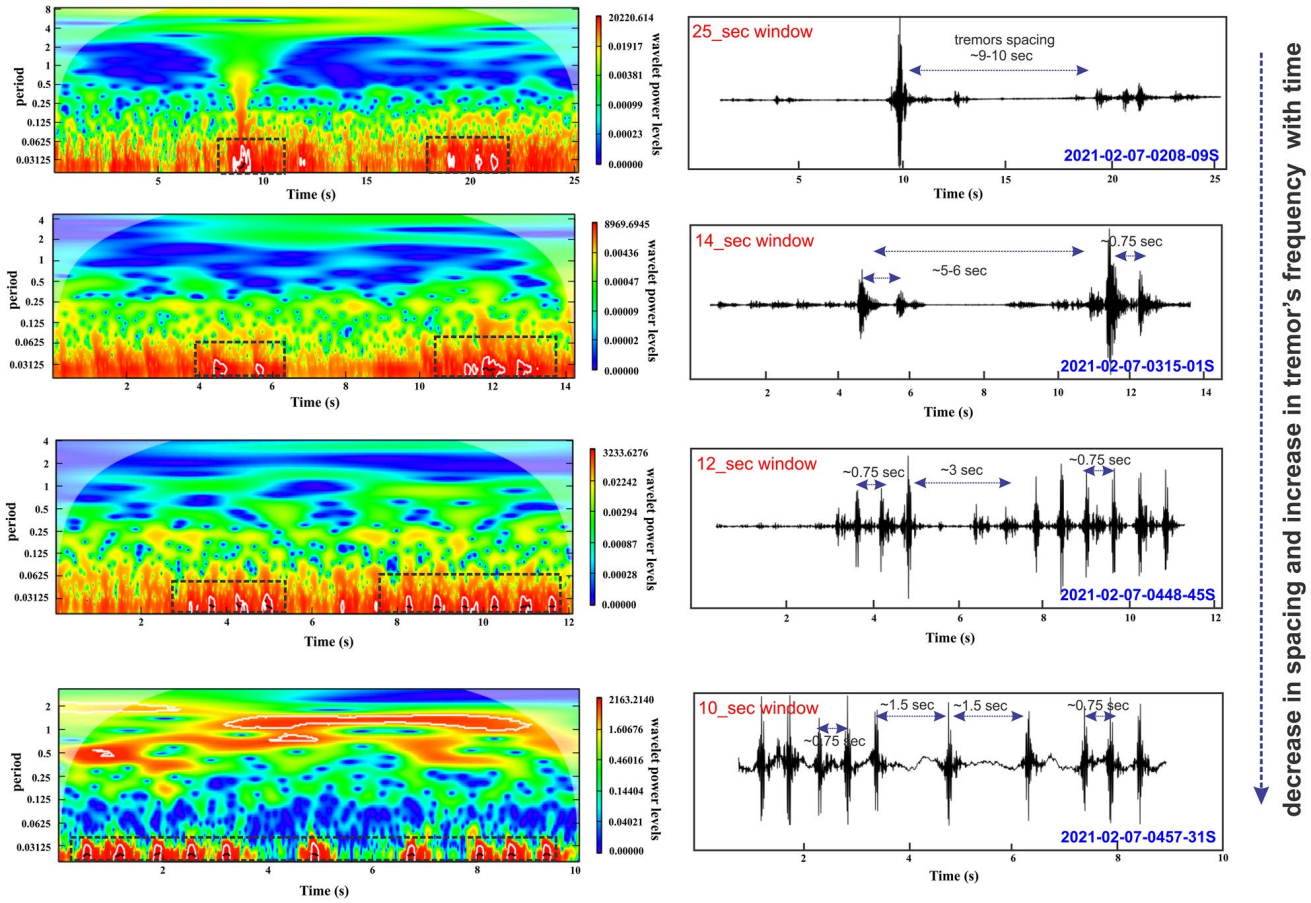
Frequency-time analysis for continually occurring precursory tremors. Seismic signals and continuous spikes were recorded in the nearest TPN station before the main release. The black dotted rectangles at the bottom of the colour plot denotes the continuation of high frequency (> 32 Hz) impact that was dominated throughout the seismic waveform window. The waveform spectrum shows increase in seismic events with decrease in spacing with time before main detachment. The whole spectrum of continuous precursory signals is also analysed for seismic window (Fig. S5).

A seismic network close to the source can detect the unfelt activities which needs a careful investigation in a broad range of frequency–time domain. As elastic wave travels in the material and propagates through different properties of the medium which resist or saturate the energy i.e. amplitude decay with distance from the source^13,14^. The present work investigates the prior distribution of acoustic energy in terms of seismic signals/tremors generated from the source of detached wedge. The detection criteria and selection of tremors is based on high signal-to-noise ratio (SNR) with threshold maintained (SNR > 10), amplitude variation, correlation coefficient and frequency time analysis (Figs. 2, 3 and Fig. S3). We have observed a long hour’s trend of continuous harmonic tremors with high frequency signals (10–35 Hz) prior to the main detachment (Fig. S4B). We have also detected the clusters of precursory signals in 4 h long east component at the nearest TPN (Tapovan) station. The cumulative number of selected tremors increases abruptly after ~ 3:10 h which indicates the advancement in dynamic phase of activity (Fig. S4C). It is acknowledged that various ice-quakes and land-quakes generate gliding harmonic tremor with a frequency that varied between 1 and 35 Hz and these events reflects the fracture propagation and subsequent flow of water in the cracks or fractures^40,41^. The spatio-temporal distribution and continuous propagation of seismic tremors are observed only in the nearest station (12 km from the source). It is detected that the high frequency tremors get quickly decayed/attenuated with distance than the low frequency signals^42–44^. We have also noticed that high frequency seismic tremors got saturated and restricted only to seismic station in proximity of the source zone. Whereas, a portion of precursory seismic tremors was weakly recorded in (GRGA) Garurganga seismic station (next to TPN station). The quantification of tremors in TPN station follows the given threshold condition of correlation coefficient trace (Fig. 2). The red dashed line implies the threshold of tremors selection above which precursory tremors are detected for similar events generated from the same source. Based on P and S phases of tremors, we locate the epicenter of these long distributed signals within the source of main detachment (Fig. 2B). The precursory tremors of > 32 Hz indicates average increase in tremor’s amplitude with time (Fig. 3 and Fig. S5). The spacing between two subsequent seismic tremors decreased with time that probably indicates the static to dynamic changes in surficial activity within the source. In the recent wedge failure mechanism, three phases of time-dependent creep were observed i.e. an initial accelerating period of strain advancement termed as transient creep, followed by static to dynamic changes within multiple weak zones, and resulting into material failure by the localization of significant deformation termed as the accelerating creep”. The recorded precursors of high-frequency continuous seismic tremors imply the active source dynamics, fracture propagation and subsequent flow of water in the cracks due to internally driven force in the weak section of the rock/material. This suggests that the surroundings of weak wedge in which an enormous volume of material is progressively added for the avalanche before reaching its threshold of material failure and simultaneously increase in tremors amplitude and signal duration. Alternatively, it infers that a significant amount of added material exerts a strong force within the weak zone and generates a strong signal-to-noise ratio for a longer time. The seismic records of main detachment and associated rock slide in the peak time of material failure can be seen in 1–5 Hz frequency. This is evidently recorded in all the Himalayan seismic observatories and in its periphery also. But other associated precursory seismic signatures recorded in high frequency range (10–35 Hz) were restricted only to the close proximity of the source or release zone. We observed some precursors of similar characteristics evidently recorded in all three close seismic observatories at midnight. The observed characteristics of precursory signals indicate the detachment and crack advancement (1st phase of wedge failure mechanism) was ongoing at midnight after that continuous static to dynamic activity had started from early morning. We have also investigated that the dynamicity of tremors continued after the main detachment (rock-ice avalanche) for some time till ~ 05:03 h (UTC). We suggest the seismic tremors after the main event as a relaxation time to recover the stability of the material within the source zone. Here, our observations highlighted the importance of background noise characterization and high frequency seismic tremors which got sharply saturated and recorded only in the nearby seismic observatory.

## Chronological sequence of events

**Figure 4:**
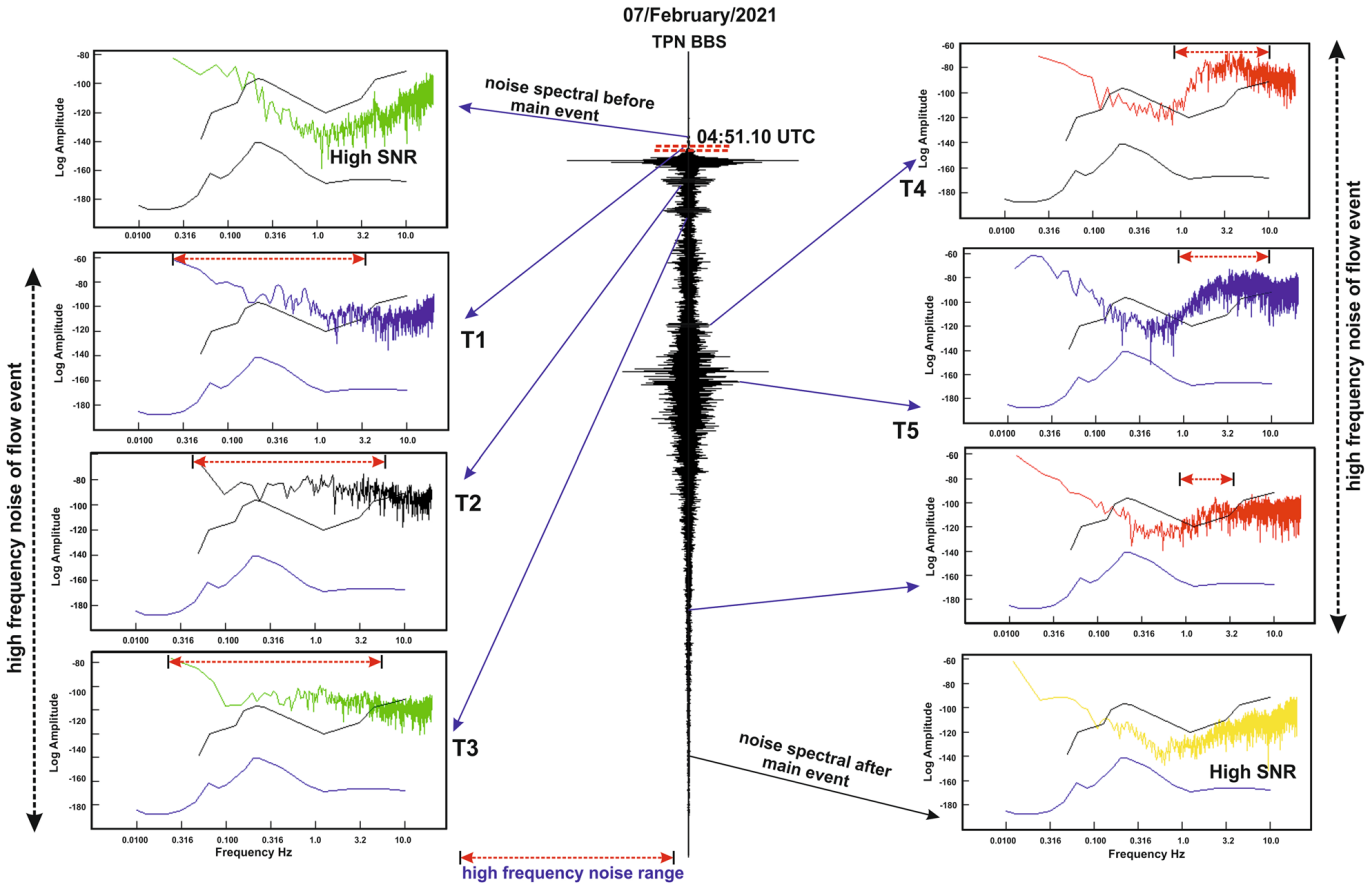
Seismic waveform spectrum and their noise PSD analysed before, during and after the main event. The events are recorded at nearest TPN station and compared with the Peterson model. During the initiation of the main event and successive impacts of extreme flow events, the high frequency noises are evidently recorded in the nearest station (TPN).

**Figure 5:**
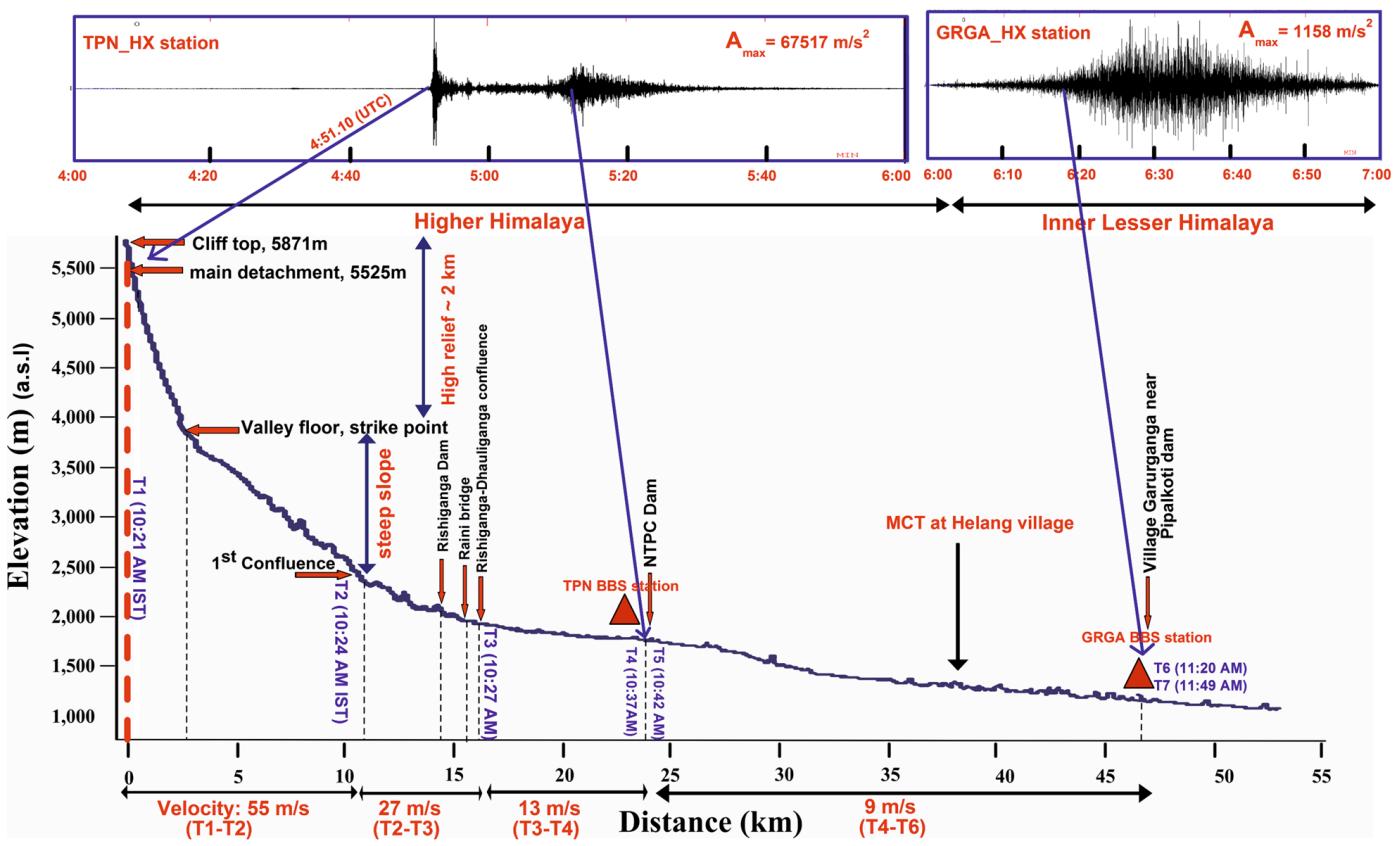
A longitudinal-elevation profile of debris flow with distinctive flow dynamicity is shown along the river path in Rishiganga–Dhauliganga valley. The profile shows the successive path of devastation with a chronological sequence of events and change with elevation above mean sea level. The seismic waveform (5–10 Hz) depicts the impacts recorded in two close stations. TPN observatory depicts the initial phase of the main detachment with subsequent events and GRGA observatory shows the nearest impact recorded in the Garurganga village and Pipalkoti dam.

**Figure 6:**
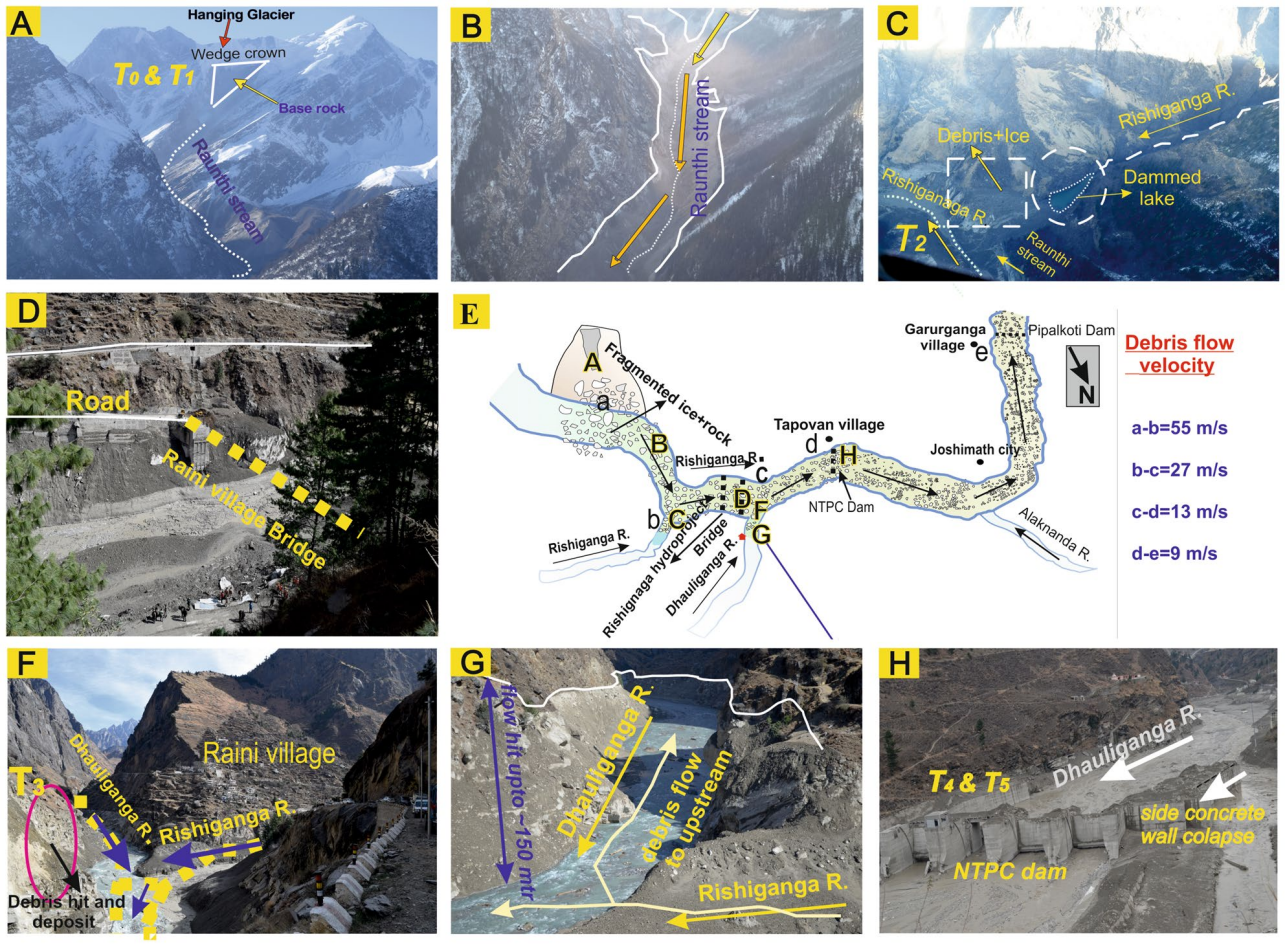
Flow dynamics and associated impacts are shown in aerial-field photographs (aerial survey immediately after the disaster. (**A**) Avalanche release zone, dotted line represents the path of rock-ice avalanche material along the Raunthi stream, (**B**) narrow steep slope channel shows the hit of debris material (white solid line) along both the banks of the stream, (**C**) dammed lake formation at the confluence of Rishiganga River and Raunthi stream, (**D**) damaged bridge at Raini village, (**E**) schematic diagram showing the successive path of debris flow with flow velocity, (**F**, **G**) evidence and signature of impactful hit and back thrusting at the confluence of Rishiganga and Dhauliganga River, (**H**) deposition of slurry material and associated impacts at Tapovan dam.

We found a good agreement of time series chronology of rock failure and flow events with seismic signals. The selection of seismic impacts follows amplitude variations, SNR and noise power spectral density (PSD) of rock-ice avalanche and subsequent debris flow (Figs. 4, 5 and Fig. S6, Table S1). The recorded seismic signals related to the avalanche are based on changes of the momentum associated with the velocity and steep elevation. The subsequent flow event and its impact recorded in seismic waveform spectrum were verified with available videos and field photographs (Fig. 6). The noise PSD analysis indicates the presence of high frequency noise during the wedge failure and subsequent flow of flash flood that is evidently recorded in nearest seismic station (Fig. 4). The seismic noise of flash flood got normalized after the complete scenario of debris flow (approximately after 05:30 UTC) and evidently verified in the Peterson noise model. Considering the downward steep angle and narrow valley, the velocity of debris flow varies continuously with an increase in avalanche mass and hits the obstacles with large force.

**Figure 7:**
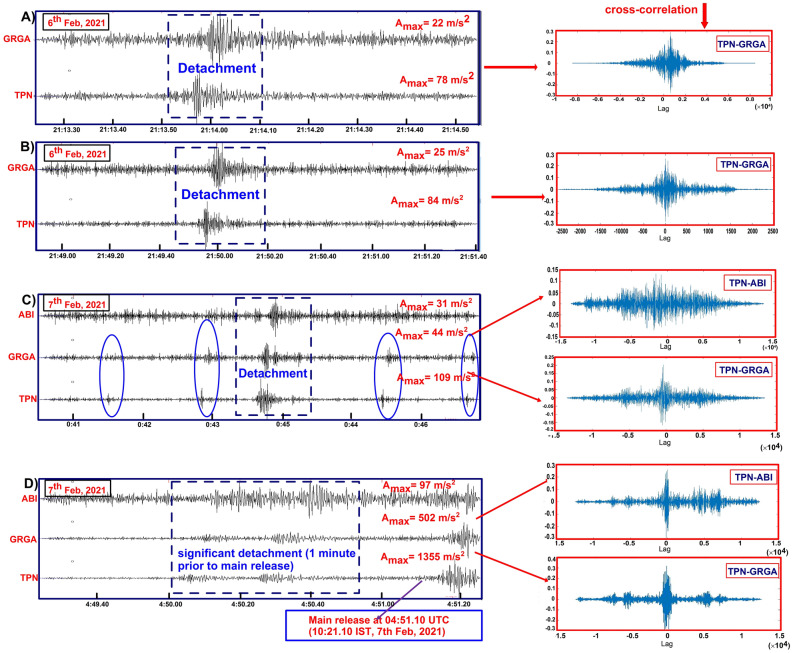
Waveform cross-correlation between different stations denoting the nucleation phase (weak zone advancement and pre-detachments) had started a day before avalanche release and correlated perfectly. It is seen that all the impacts are first recorded in the TPN observatory, which is near to the source. Waveform cross-correlations (**A**–**D**) are depicted for the blue dotted rectangle. (**C**) The blue elliptical circle shows the weak/small detachment recorded in two nearby observatory. (**D**) Significant detachment or weak zone advancement (T_0_) just 1 min prior to the main release/detachment (T_1_) can be seen in three nearby seismic stations with perfect cross-correlation of seismic waveform.

The unfelt rock-fall activities associated with crack development had started prior to main avalanche, which was recorded in the broadband seismic stations close enough to the source. The high-frequency seismic signatures were recorded and can be seen in a frequency range of 10–35 Hz. We found that the amplitude were generally higher for the horizontal components of the recorded seismic spikes/tremors attribute, which we understood as influence of surficial dynamics. We have observed several detachments (seismic tremors) before the main release recorded in three stations, which imply that the source is identical using the wave cross-correlation method (Fig. 7). These seismic tremors were first recorded in TPN observatory which is much closer to the source and indicates crack development and associated fall of rock/ice fragments onto the valley floor from the avalanche release zone.

On 6th Feb 2021, several impacts were observed at two nearby seismic observatories. Later on 7th February 2021, the impact was observed in three stations with the increase in amplitude of seismic spikes (Fig. 7). The increase in such amplitude of seismic spikes indicates weak zone advancement (also recorded in 3rd station) and transition from static to dynamic process that enhances the nucleation phase preceding the avalanche release (Fig. S4). Variations in tremor activities on different timescales are identified based on amplitude variations and time gap between successive tremors. It is noticed that the amplitude of tremors increases and time gap between tremors decreases with time. It is also suggested that similar quasi-static to quasi dynamic activity led to a nucleation process to trigger the weak zone, which further results in significant slip in landslide, earthquake and avalanche^17,45,46^. The vicinity of failure slope is bounded by two sets of joints and a foliation^37^, hence the static to dynamic nucleation process further weakens the structure along the joints and foliation that led to a wedge type failure. Before the avalanche was released, we found significant continuous high amplitude spikes (almost 2:30 h prior to the avalanche release) at the time of full sunrise ~ 7:30 a.m. IST. It is evidently seen that high-frequency seismic signatures (impact of weak zone advancement and acceleration of nucleation phase) were continuously recorded at the TPN observatory only (Fig. 3 and Fig. S5). We also see that the movement in an unconsolidated regime and its progressive melting driven by external forcing under large superficial blocks does not generate a similar pattern of continuous seismic wave or is not significant enough to record^47^. These superficial activities are less energetic with a longer duration than the internally driven forces (i.e., creeping, crack and fracture advancement). Hence the recorded high-frequency continuous seismic spikes imply the dynamics of internally driven force in the weak section of the rock/material.

The active signatures in seismic waves correspond to time-varying forces that are caused by the acceleration and deceleration of a sliding mass interacting with the Earth's surface. The crown of wedge failure developed successive changes in scarp with time from 2015 to 2021^37^. This implies that the failure planes bounding the detachment developed years before the main release, which needed to be activated. Along with the successive changes in wedge scarp, maximum inward depth is also found in the vicinity of crown of the wedge. Hence it is suggested that the failure had started from the top of the wedge with continuous crack development, evidences of which are recorded in TPN station in high frequency prior to the main release. The advancement of weak zone that supports disintegration of ice/rock fragments was continuously active from 2:08.09 to 5:03.10 (UTC, 7th Feb 2021) from the wedge crown at 5525 m asl and fall into the valley channel at 3600 m asl. There was a sudden increase in the amplitude of seismic spikes from 03:10:28 (UTC) to the time of wedge failure. It is also suggested that the seismic spikes prior to the main release are the signals of development and expansion of cracks in the rock-ice mass of weak zone (crown edges), which results in the fall of rock and ice fragments. Our findings suggest the crack development and enhancement of weak section in the wedge were responsible for this catastrophic event. After the main release, we get similar continuous seismic spikes that ended suddenly at 5:03.10 UTC (10:33.10 IST), and can be considered as relaxation time to maintain the stability in the source zone (Fig. S5). The 35° degree slope with more than 2000 m relief yields the certainty of disintegration of ice/rock fragments into smaller pieces. We believe that the distribution of disintegrated ice/rock fragments prior to the main release developed a low friction path, which enhanced the velocity of avalanche debris during main release along the steep slope. The huge elevation difference (more than 2000 m relief) supports the temperature variability and prominent disintegration of fragments of the falling material prior to main release and lead to melting of snow and ice which enhance the slurry-water composition during the main release.

According to some villagers and local workers, they heard sounds (considered it to be rock blast) at ~ 2:30 a.m. IST 7th February (~ 20:30 UTC, 6th Feb) and at the time of the main release on 7th February 2021. Although both sounds were significant enough, however the former one was less significant compared to the latter one. Several considerable imprints of amplitude spike were recorded only in proximal station at midnight around 2:30–3:30 a.m. and nearly 1 min prior (4:50.06 UTC, Table S1) to the main release (04:51.10 UTC) (Fig. 7 and Table S1). These imprints of amplitude spikes have been correlated, and the results indicate similar source of events. We interpret these signals as the nucleation phase of material failure and initiation of a significant wedge failure that had started nearly 1 min earlier and triggered the avalanche. The avalanche was generated from a huge wedge-shaped mass consisting of rock loaded with overlying ice that ruptured and fell down into the steep slope of Raunthi valley^35,36^. A huge amount of energy and noise was released from the breakdown of ~ 27 × 10^6^ m^3^ wedge after hitting the valley from the source^35^.

## Impact evaluation from seismic and field evidences

In the catastrophic event, the impact of a flash flood was mainly constrained to the modern structures i.e. two hydropower projects, bridges and roads along the river path. The debris and huge boulders were distributed all along the river channel. The field investigation shows the sequence of impacts which is still present (as on date July 2021; Fig. S2) as a deposit of debris, which went up to 150 m high, with large boulders distributed on both banks along the river channel. The field observation indicates that the avalanche caused a sudden impact and horizontal rupture of dams and bridges located in the path of the Rishiganga and Dhauliganga Rivers. These structures offered a resistance and then collapsed after the accumulation of a large amount of debris, boulders and slurry water. This impact affects the entire structure along its foundation with an efficient coupling into the ground. The horizontal dislocation and breakage of the modern structures are capable to generate a seismic signal strong enough to be recorded in the nearby seismic observatories. The rupture of the structures along its foundation yields efficient coupling into the ground that generated seismic signals. The question arises here: can these ruptures and dislocation be linked with the change in seismic amplitude pattern, so that we can know the exact time of destruction using nearest stations? We are convinced that the answer to this question is “Yes” based on the observations of local people, real-time synchronization of a video shoot (breakage and debris flow time), telephonic conversation time between the locals at the time of collapse. The time of verified evidences and observations is truly linked with the time of change in seismic pattern (surface noise pattern). High seismic amplitude is considered to represent the onset time when debris flow changed its path, either through change in slope or through obstacles/turn in its path. The observed impact time (T_0_–T_7_) are based on difference in seismic amplitude variation and its onset time, high SNR, noise PSD, advancement of destruction along the hitting obstacles (video captured by locals) and our investigation to approach the dam workers and captured the screenshots of phone call timings between them at the time of evacuation from the tunnel (during the hit at Tapovan dam) (Figs. 4, 7 and Fig. S6). The relative abrupt change in seismic amplitude (T_0_–T_7_) verified with the field evidences (change in altitude, valley width, debris flow, hitting impacts and obstacles). Based on cross-correlation method between waveforms of different seismic stations, it is clearly seen that the T_0_ is marked as the significant weak zone advancement just 1 min prior to the main release that is evidently recorded in all three nearby seismic stations (Fig. 7). T_1_ is evidently recorded in all the stations which are deployed along the extent of Himalaya^35,38^. The time series T_2_–T_7_ is also evidently recorded with different amplitude variation, SNR and observed noise PSD (Fig. 4 and Fig. S6). Here analysis of noise PSD indicates the presence of very high frequency noise during subsequent flow of flash flood that is clearly recorded in nearest seismic station. Followings are the impact of events verified with the seismic signals based on the change in momentum, altitude and obstacles along the river path in the downstream.

- **T**_**0**_: Crack or fracture advancement at the weak zone in crown of the wedge and associated weak detachment; 4:50.06 UTC (10:20.06 a.m. IST).
- **T**_**1**_: Avalanche release from the detachment zone and impact on Raunthi valley floor; 04:51.10 UTC (10:21.10 a.m. IST).
- **T**_**2**_: Impact of debris material that blocks the River Rishiganaga (large curvature of river path or a sharp turn to west); 4:54 UTC (10:24 a.m. IST).
- **T**_**3**_: Impact of debris material that damaged Rishiganaga hydro project (HEP), bridges and then hits Dhauliganga at the confluence point (goes to upstream and comes back as the 2nd wave with high flow intensity); 04:57 UTC (10:27 a.m. IST).
- **T**_**4**_: 1st wave of debris material that hit the Tapovan (NTPC) HEP; 05:07 UTC (10:37 a.m. IST).
- **T**_**5**_: 2nd wave of debris material that hit and damaged Tapovan (NTPC) hydro project; 05:12 UTC (10:42 a.m. IST).
- **T**_**6**_: 1st wave hitting impact at Pipalkoti dam near Garurganga village; 05:50 UTC (11:20 a.m. IST).
- **T**_**7**_: 2nd intense wave impact at Pipalkoti dam; 06:19 UTC (11:49 a.m. IST).

We found several large-scale impacts and associated changes in flow momentum that were recorded in the TPN seismic station (Fig. S6) in Rishiganga–Dhauliganga valley. The 1st wave of slurry material hits the Rishiganga–Dhauliganga river confluence at 10:27 a.m. (IST) and most of the debris flow material was back thrusted to 1.5 km upstream along the Dhauliganga River (Fig. S7). Later on, the release of this back thrusted debris and water caused the 2nd wave towards downstream leading to destruction of a temple located at the right bank of the River Dhauliganga near the confluence with the Rishiganga River. The Tapovan dam withstands for some time against the forces applied by the 1st wave of the debris flow. During the accumulation of slurry material of 2nd wave at the sidewall, the momentum was heavily transferred directly to the foundation and other associated structures and subsequently transferred to the ground. The high amplitude signals of noise pattern are clearly visible in the TPN seismic observatory along with the noise of debris flow which indicates the collapse of the barrage gate at Tapovan dam. The maximum impact of debris-slurry flow was observed at the Rishiganga project and Tapovan dam. We have calculated the flow dynamics of the debris flow with velocity variation against the change in slope of the longitudinal path of the river (Fig. 5). Furthermore, we have also observed the significant seismic spikes of debris flow when it reached Pipalkoti dam near Garurganga village. The relative seismic spikes were observed in the GRGA (Garurganga) broad-band seismic (BBS) station, which is located at Garurganga Village. The timing of seismic spikes with its closest impact on dam was verified with the officials of the Pipalkoti dam. We have found a drastic decrease in flow velocity after the devastation of Tapovan dam because of the relatively gentle slope and wider river channel, after that the intensity of flow almost got normalized in the downstream of Alaknanda River.

## Flow dynamics

Debris flow, a frequent phenomenon in the high terrain mountainous regime, consists of steep slope front (head side) with high sediment proportion and huge boulders, followed by additional fluidal and turbulent slurry (at tail)^36^. The flow front of recent rock-ice avalanche consists of high debris load along with the rock-ice material, which hit the surface of obstacles with large force that can be detected with high seismic amplitude in nearby seismic stations. Later on, the same obstacles were struck by the fluidal and turbulent slurry, which is recorded as high frequency noise in the seismic stations but not recorded as change in high seismic amplitude. Hence, we have identified and correlated the onset of high amplitude signals with the timing of successive obstacles struck by the flow fronts at different time scale and different altitude along the river path in the downstream. The destruction of modern infrastructure due to debris flow usually leads to disastrous episodes. The estimated flow dynamics of recent debris flow is verified based on the chronological sequence of significant surface impacts with GPS synchronization time (± 0.1 ms) recorded in close seismic observatories. A significant landscape relief of more than 2 km and the average inclination of ~ 35° slope enhanced the flow of debris material with high velocity. This energetic flow along the plane of movement enhanced the frictional heating of the ice that allowed the variation in flow characteristics and successive increase in fluidal proportion with sediments and ice block in the down valley^48^. The successive natural and modern obstacles impact during the debris flow was recorded in the proximal stations with distinctive phases along with the longitudinal river profile (Fig. 5).

We present different velocities of debris flow, based on the recorded and estimated seismic timing of hitting large flow obstacles. It originated with high velocity ~ 55 m/s from the source (T_1_) to Rishiganga–Raunthi stream confluence (T_2_). The confluence of the Rishiganga–Raunthi stream (11 km away from source: longitudinal river path) was the point where debris flow took a sharp turn towards the Rishiganga project (Fig. 6C). At this confluence point, we have found a thick deposit of debris owing to deceleration and sharp turn of the flow, which blocked the flow of the Rishiganga River and formed a dammed lake. The velocity decreased to ~ 27 m/s (verified with available videos) from the 1st confluence (T_2_ in Fig. 6C) to 2nd confluence of Rishiganga–Dhauliganga (T_3_ in Fig. 6F). We have found the large reduction of frontal velocity down to 13 m/s from 2nd confluence (T_3_) to Tapovan dam (T_4_ in Fig. 6H). The reduction in flow velocity was due to impoundment or concrete wall obstacles behind the Tapovan dam. The 2nd wave of debris flow destructed the left side wall of the Tapovan dam at time T_5_. The calculated mean discharge ranges in between ~ 8200 and ~ 14,200 m^3^ at the Rishiganga hydropower project and ~ 2900 to ~ 4900 m^3^ at the downstream of Tapovan dam^35^. Later on, the velocity decreased to ~ 9 m/s and almost normalized before reaching Karanprayag, which is the confluence of Alaknanda and Pindar Rivers (45 km away from Pipalkoti dam in downstream).

## Hazard mitigation: a necessary aspect of early warning system

After the recent deadly avalanche, the government authorities have installed a automatic water level recorder (AWLR) with siren, ~400 m upstream of Raini village. The main objective of this installation is to detect any unusual rise in water levels of Rishiganga River due to the artificial lake formed at the confluence of Rishiganga-Raunthi streams and warn the population for instant mass evacuation. Unfortunately, we are still facing a challenge to understand the physical processes involved in the dynamic avalanche ruptures. Due to lack of continuous real-time monitoring, it is hard to detect the early stages of failure in the proximity of the source that involves a chain of processes. The identification and analysis of early stage activities recorded in seismic stations highlight the consideration of precursory signals. To protect the population against such deadly events, scientific and government authorities should plan a multi-parameter early warning system that is the only efficient way in reducing the impacts of the disaster.

In the present study, we have demonstrated that the seismic footprints may be a promising approach for the investigation of such disasters and the possiblity for development of an early warning system. Based on the potential demand for early warning aspects and techniques to reduce the risk of disaster and loss of life, our findings suggest that continuous real-time seismic monitoring and decision making can be utilized for warning prior to the main incident. Deep learning of seismic waveform with significant long-term changes in a high-frequency domain can lead to understanding the characteristics of movement within the periphery of the source zone. The study shows that pre-signatures of continuous nucleation advancement of rock motion for ~ 2:30 h was active prior to the avalanche release. We also found a significant sequence of high amplitude waveform patterns corresponding to creeping and crack development in crown of the wedge. It is noticed from the records of seismic stations that the prominent destruction and human casuality turn out after ~15–17 min of the main release of the avalanche. This could have provided enough time for mass evacuation and hazard mitigation. After this event, we have been continuously working on multiple parameters to characterize and demarcate the potentially dangerous zones in the vicinity of glaciers. The present findings suggest that continuous real-time seismic monitoring would be significant investigating parameters for the detection of prior unfelt natural activities, which may lead to deadly events. For accuracy and to avoid false alarms, we need a proper integrated network of highly sensitive hydrological, meteorological and seismic (good azimuthal coverage) observatories. Hence, the identification of recorded pre-signatures of seismic signals in real-time monitoring could be used as a warning to mitigate the risk related to natural hazards.

## Conclusion

We have analyzed seismic data of a deadly rock-ice avalanche (07 February 2021), its precursory signals and chronological advancement/impacts of dynamic events to reconstruct the entire scenario of devastation in Rishiganga–Dhauliganga valley of Chamoli district, NW Himalaya. The seismic stations were exceptionally close to the source and record the high quality data with high  signal to noise ratio. A detailed analysis of recorded seismic data shows the continuous high frequency signals that were generated ~ 2:30 h before the main release of avalanche. The identification of pre-signatures with  simultaneous increase in tremors amplitude and signal duration in seismic waveform spectrum implies that the vicinity of weak wedge was gradually creeping with the expansion of cracks and fracture propagation before release of rock-ice avalanche. The exact time of main detachment first recorded at the TPN station at 04:51:10 UTC, which was 12 km away from the source zone. The continuous creeping with different frequencies, SNR’s, amplitude variation indicate static to dynamic phase, which triggered the weak wedge shaped avalanche of ~ 27 × 10^6^ m^3^ mass. It has been recognized that numerous cracks had started to open after 2015 and led to successive advancement of weak zone near the crown of the wedge failure (source zone). A gradual process of crack development had started years before the main activity of February 2021, which just needed to be activated from steady to dynamic phase for sudden failure. A chronological reconstruction based on recorded seismic signals and field evidences of the recent devastation shows the energetic impacts in the downstream. The mixture of rock and ice travelled with extremely high velocity flow front (initiated with ~ 55 m/s speed) from the source (average 35° slope) towards the modern structures that collapsed with big impacts. The estimation of different velocities are evidently based on the onset timing (GPS accuracy ± 0.1 ms) of abrupt changes in seismic amplitude and calculated distance from the source to each obstacle that was recorded in TPN seismic station. The flow dynamics, narrow river channel with high slope and mixture of huge boulders with slurry material played a prominent role for high magnitude impacts. The flow front got normalized after Pipalkoti hydro project that was ~45 km away from the source of Raunthi peak.

Our observations based on seismic findings show the warning potential of seismic monitoring for the avalanche. A real-time monitoring of sliding and creeping of fracture and crack advancement is needed to know the degree of criticality and instability of source zone. The main destruction in Dhauliganga valley led to huge casuality in Tapovan dam that happened 15–17 min after the initiation of rock-ice avalanche. Had there been an early warning system in place, the study shows that there was enough time for mass evacuation. This study further indicates that the high quality data with close dense seismic array is needed for monitoring the potentially dangerous zones. This approach would delineate the dynamics and degree of criticality of natural hazards of the region and can thus improve our attentiveness for significant hazards by providing timely warning for any disasters. Furthermore, we suggest the deployment of dense seismic network with other early warning parameters in the periphery of existing potentially dangerous zones would increase the efficiency of unfelt event detection, processing of signals and development of early warning system. This would also lead to a timely identification of new potentially active zone, which can create a critical situation in future. Hence, an integrated early warning perspective with a capability of practical response, communication, education and awareness would provide an effective approach for hazard mitigation.

## Supplementary Information

Supplementary Information.

## Data Availability

In the present article, all the data have been used from the observatories run by the Wadia Institute of Himalayan Geology installed near the source. The related seismic data is available upon request from corresponding author/Director, Wadia Institute of Himalayan Geology, Dehradun, India.
