# Supplementary material for: Potential seismic precursors and surficial dynamics of a deadly Himalayan disaster: an early warning approach
Source: Sci Rep. 2022 Mar 8;12:3733. doi: 10.1038/s41598-022-07491-y (PMC8904521; doi:10.1038/s41598-022-07491-y)
Supplement: Supplementary file 1 — Supplementary Information. [file 41598_2022_7491_MOESM1_ESM.docx]

**Supplementary material**

**Potential seismic precursors and surficial dynamics of a deadly Himalayan disaster: An early warning approach**

Anil Tiwari, Kalachand Sain*, Amit Kumar, Jyoti Tiwari, Ajay Paul, Naresh Kumar, Chinmay Haldar, Sushil Kumar, Chhavi Pandey

Wadia Institute of Himalayan Geology, 33, General Mahadev Singh Road, Dehradun - 248001, Uttarakhand, India

***Corresponding Author email**: kalachandsain7@gmail.com; director@wihg.res.in

**Supplementary material**

Text: Data and Methods

Table: S1

Figures: S1-S7

References: 49-55

**Data and Methods**

**Seismic Network**

The seismic data used in this article was investigated for avalanche impact analysis and associated precursory signals. All the seismic data arise from WIHG, with primary focus on three nearest stations to the avalanche location, which are the part of fixed seismic network of Garhwal Himalaya and continuous seismic data are being recorded since its deployment in 2007. The stations are located at the distance of 12 km (Tapovan (TPN) station), 28 Km (Garurganga (GRGA) station) and 45 Km (Adibadri (ABI) station) from the source. For the suitability of the seismic site, the recorded noise spectrum has been verified and fits in the Peterson noise model. Much attention was paid to detect the pre-signature of significant changes in waveform before the avalanche and time series of devastations after the avalanche. The data were generated and processed at the earthquake data processing center of the WIHG, Dehradun, India. This data was analyzed using the SEISAN software package^49^. Continuous seismic waveforms at several stations of the WIHG network in the Garhwal Himalaya were visually screened in the SEISAN window in different frequency domain. Although other seismological agencies analysed data from the seismic stations which are located very far from the source of the release zone also found the record of wedge failure and slip^31,38,50^. But the WIHG is the only agency that has deployed a fixed network of highly sensitive broadband seismic (BBS) stations fortunately at the proximity to the source of the avalanche release zone. The BBS stations are deployed with Trillium-240 sensors and high dynamic range (>138 db) with Centaur data acquisition system (DAS). Each station records continuous data in the standard frequency bandwidth range of i.e. 0.004-50 Hz, where the digitization of 100 samples per second with three components is shown in the waveform window. High-accuracy GPS (± 0.1 mSec) synchronies the DAS clock every minute.

**Selection Criteria for Tremors**

From the seismographs recorded by the nearest stations, we observe a clear cyclic high frequency seismic energy, hereafter called the precursory tremors generated from the source of Raunthi peak. To quantify the evolution of precursory tremors we fixed and maintained a selection criteria aimed to detect and evaluate the dynamicity of the source. We applied the algorithm of short term versus long term amplitude evaluation to detect the onset time of the precursory signals. Over 4000 events are detected based on the trigger threshold from the ratio of short term (STA) and long term amplitudes (LTA). However, the adopted trigger threshold of 10 nicely detect these events but there may be a scope of uncertainty of false detection or skipping of few events. To assess the uncertainty in the detection, we extracted events using variable trigger threshold of STA, LTA ratio between 5 and 20. 4723 events are detected of threshold ratio of 5, number of events decreases with the increase of ratio with 3412 events at ratio 20. The average number of events are 4010 with standard deviation of ±333 tremors (i.e. ± 8 %). The trends of events occurrence and their detection based on variable thresholds are observed which indicates that detection has good resemblance for the threshold ratios between 8 and 12 with a difference of ~200 events with an error of nearly ±5 percent. Hence we have selected those tremors which follows the threshold of SNR ≥ 10 which is recommended for a good signal to noise ratio and less detection error for seismic tremors. A selection node for tremors is based on SNR threshold (SNR>10), amplitude variation, waveform cross-correlation, events detection simulation, correlation coefficient and manually verified in seismic waveform spectrum (Fig. 2, 7 and Fig. S3, S4). The selection node varies between 0 and 1, we convinced with criterion that follows to 1 and avoids those tremors which maintained to 0. Data quality was further manually checked and removed the few seismic tremors in presence of poor SNR. Based on these selection criteria we observed the subsequent increase in tremors amplitude prior to the material failure/detachment and dropped sharply afterward which signifies the temporal variation of the emitted energy in different frequency time domain. A sharp increase in cumulative numbers of precursory tremors after 03:10 Hr indicates the potential changes in dynamic phases of source. At midnight of 7^th^ February 2021, based on amplitude variation and cross correlation, we found few significant detachment (villagers also heard sounds) near to source recorded in three nearest stations. Whereas high frequency (10-35 Hz) seismic tremors are recorded and evidently observed only in one seismic station which is very close to the source. The midnight activities can be seen in 10-25 Hz, while a very high frequency up to ~35 Hz is predominant in seismic records at ~2:30 Hr prior to the main activity. A cumulative number of tremors and amplitude variation are more variable and intensified (also shown in frequency time analysis plot, Fig. S6) after ~3:00 Hr UTC (Fig. S4) which we interpret as increase in dynamic activity in the source of weak zone.

**Correlation Coefficient**

The quantification for evolution of precursory tremors is based on correlation coefficient using coherence-based method^51^. The objective of this process is to exploit the similarity between events, which were checked for 4 Hr window (before this time no amplitude variations and tremors is observed in waveform spectrum), before and after the main event. A standard tremor based on high SNR has been selected for moving window correlation (Fig. 2). When the correlation coefficient (similarity of the events) is found above the threshold, a precursory is detected. The results based on correlation coefficient method provides good number of detected precursory signals. Furthermore, the similarities have been verified with SNR threshold to refine the selection node process for detected precursory tremors. We observed an abrupt change in the elevation difference of correlation coefficient (8 times the median deviation of correlation coefficient trace) after ~3:10 Hr UTC (07/02/2021). We interpreted this as the change in static phase to dynamic phase of creeping and crack/fracture advancement.

**Wavelet analysis**

Wavelet analysis is a powerful tool to study the periodic phenomenon in the time series particularly in the presence of potential frequency changes in time. We have applied continuous wavelet transformation (CWT) that provides localized spectral description of the analyzed dataset. For the computation of CWT, we have used an open source package named as ‘WaveletComp’ (R language)^52^. After transformation this tool converts the one dimensional time series into two dimensional color map that represents the evolution of scales and frequency with time. Mathematically, the wavelet can be represented as a small wave that is restricted in time. It is necessary for wavelet function ψ(t) to justify the following conditions (i.e. the function must have zero mean and finite energy):

$\int_{-\infty}^{\infty} |\psi(t)|2dt < \infty$ and $\int_{-\infty}^{\infty} \psi\left( t \right)dt=0$ (1)

In general, the transformation of wavelet represents the correlations between wavelet function and time series signals, which finally results into wavelet coefficient contour map (also known as scalogram) that follows the criteria of justified confidence level in different scales. The squared modulus of CWT is defined as the wavelet power spectrum (WPS). Further details of the methodology of CWT can be found in the paper by Torrence and Compo^53^. We have applied the frequency-time analysis for signal processing using time-frequency representation (TFR) and wavelet transformation. The faded region above the inverted U-shaped curve (Fig. 3 and Fig. S5) indicates the cone of influence (COI). The cone of influence is shown on each wavelet power spectrum (WPS). Inside the COI, the wavelet power spectrum is unreliable and therefore the results in this region should be used with caution. We observed high frequency wavelet signals below the inverted U shaped curve, hence the results is reliable with the observed frequency range. This shows continuous high peak frequency which was active for more than 2.30 Hr before the avalanche release. The high-frequency signals denoted in black dots inside the contour follow the criteria of 95 % confidence level (Fig. 3) and are also encircled with white elliptical circle at the bottom of frequency-time wavelet analysis plot for 2:30 Hr window (Fig. S5). The analysis shows the continuation of the high frequency (>32 Hz) impact, which is dominant throughout the seismic waveform window, which started 2.30 Hr before the main release and sharply terminated after the complete material failure.

**Cross-Correlation**

The cross-correlation of the seismic signals has been analyzed to check the propagation and similarity of strong noise of single-source recorded in other seismic stations. A series of precursory signals are recorded and evidently seen in the waveform spectrogram in the dominant frequency range. Here we have investigated the seismic signals recorded in all three nearest stations in Rishiganga-Dhauliganga and Alaknanda Valley. The prior unfelt seismic signals/tremors related to this deadly avalanche are very well recorded in the nearest three stations with high SNR and dominant at midnight of 7 February 2021. The TPN station is closed enough to the source, which could record the emitted seismic energy from the creeping of the material. The remaining two stations recorded the high-frequency noise of nearest activity (post events) which probably corresponds to the impact of slurry/debris-water flow. To avoid false noise patterns and contamination related to some other anthropogenic activities, we have applied a cross-correlation technique^54,55^ inbuilt tool of Matlab (Xcorr) to check if the seismic records are from the same or different source. The cross-correlation of two discrete-time series measures the similarities or compares two time series between the axis of X and Y vector as a function of lag. A local noise of anthropogenic activity is hard to detect in other stations that are deployed so far. We suggests the activities (waveform pattern other than sub-surface rupture or earthquake) recorded at the same time (at midnight before the main release) in seismic stations, which are deployed near to the source are associated with dynamic activities related to the avalanche. We found the seismic amplitude is maximum in horizontal components (HHX or HX and HHY or HY), which indicates the major impacts of surficial dynamics. The high-frequency seismic signals (10–35 Hz) are identified by visual screening of continuous waveform, which was recorded in all three closed stations.

**Table-S1:** Time series of events activities prior, during and after the avalanche release

| \| **S. No** \| **Date** \| **Time (UTC)** \| **Seismic station records** \| **Activity/devastation** \| \| --- \| --- \| --- \| --- \| --- \| \| 01 \| 20210206 \| 21:13.31 \| Recorded in TPN and GRGA \| Indicates plucking of rock fragments onto the valley from the source/cliff \| \| 02 \| 20210206 \| 21:31.22, 21:34.11, 21:35.18 \| Single station (TPN) continuous seismic peaks \| \| 03 \| 20210206 \| 21:49.28 \| Recorded in TPN and GRGA \| \| 04 \| 20210206 \| 22:48.23, 2249.15 \| Recorded in TPN and GRGA \| \| 05 \| 20210206 \| 23:17.23, 23:19.50 \| Recorded in TPN and GRGA \| \| 06 \| 20210207 \| 0:43.17 \| Recorded in TPN, GRGA and ABI \| \| 07 \| 20210207 \| 1:08.25 \| Recorded in TPN station \| \| 08 \| 20210207 \| 3:53.22 \| Recorded in TPN and GRGA \| \| 09 \| 20210207 \| 2:08.09 to 5:03.10  (sudden increase in amplitude spikes after 03:10:28 ) \| Recorded in TPN station \| Indicates continuous rock motion/ creeping/ crack development and nucleation process with an efficient dynamic coupling into the ground \| \| 10 \| 20210207 \| 4:50.06 \| Recorded in TPN, GRGA and ABI \| Indicates plucking of rock or its fragments onto the valley from the source/cliff \| \| 11 \| 20210207 \| 04:51.10 \| Recorded in all three station \| Avalanche release \| \| 12 \| 20210207 \| 4:54.40 \| Recorded in TPN and GRGA \| Hit the curvature of steep slope (between Rishiganga and Dhauliganga) \| \| 13 \| 20210207 \| 04:57.05 \| Recorded in TPN and GRGA \| Collapse of Rishiganga dam \| \| 14 \| 20210207 \| 05:07.17 \| Recorded in TPN and GRGA \| Reached NTPC Tapovan dam \| \| 15 \| 20210207 \| 05:12.08 \| Recorded in TPN station \| Collapse of concrete wall of Tapovan NTPC dam \| \| 16 \| 20210207 \| 05:50.56 \| Recorded in GRGA station \| Weak seismic transient recorded \| \| 17 \| 20210207 \| 06:19.02 \| Recorded in GRGA station \| Recorded strong noise of debris flow: the time of hitting of debris-slurry material in the close vicinity of Garurganga Station \| |
| --- | --- | --- | --- | --- | --- | --- | --- | --- | --- | --- | --- | --- | --- | --- | --- | --- | --- | --- | --- | --- | --- | --- | --- | --- | --- | --- | --- | --- | --- | --- | --- | --- | --- | --- | --- | --- | --- | --- | --- | --- | --- | --- | --- | --- | --- | --- | --- | --- | --- | --- | --- | --- | --- | --- | --- | --- | --- | --- | --- | --- | --- | --- | --- | --- | --- | --- | --- | --- | --- | --- | --- | --- | --- | --- | --- | --- | --- | --- | --- | --- | --- | --- | --- |


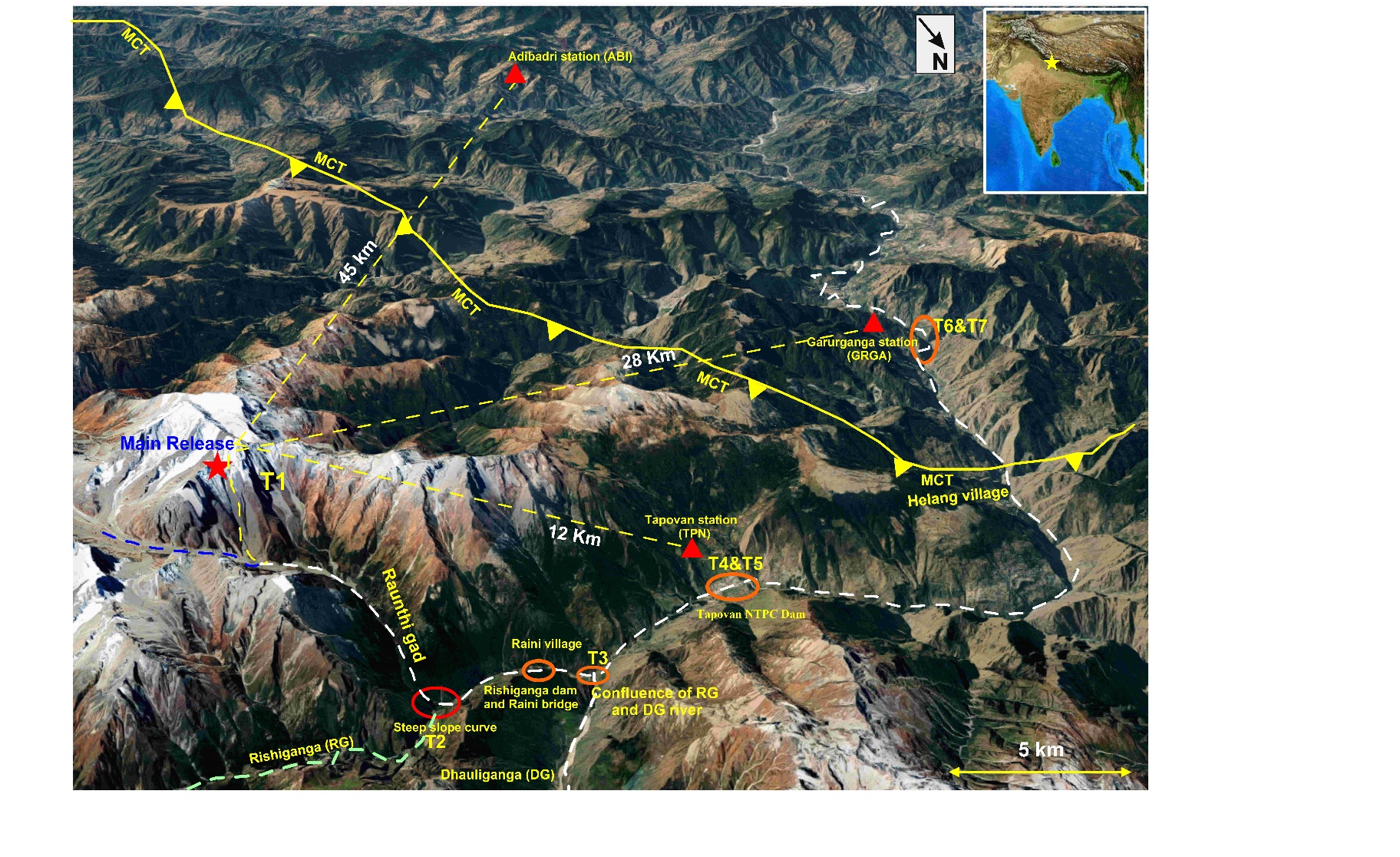


**Figure S1.** Synoptic view of the Rishiganga-Dhauliganga valley, Uttarakhand, Indian Himalaya. Red triangles show the distribution of three BBS stations, installed by WIHG and happened to be in proximity of the avalanche release zone (scarp at the weak zone of wedge crown denoted by a red star). The red elliptical circles denote major debris impacts and depositional sites in the vicinity of major Himalayan thrust i.e. Main Central Thrust (MCT). (T_1_-T_7_) denotes the successive time series of debris flow impacts recorded in real-time GPS synchronization.

**
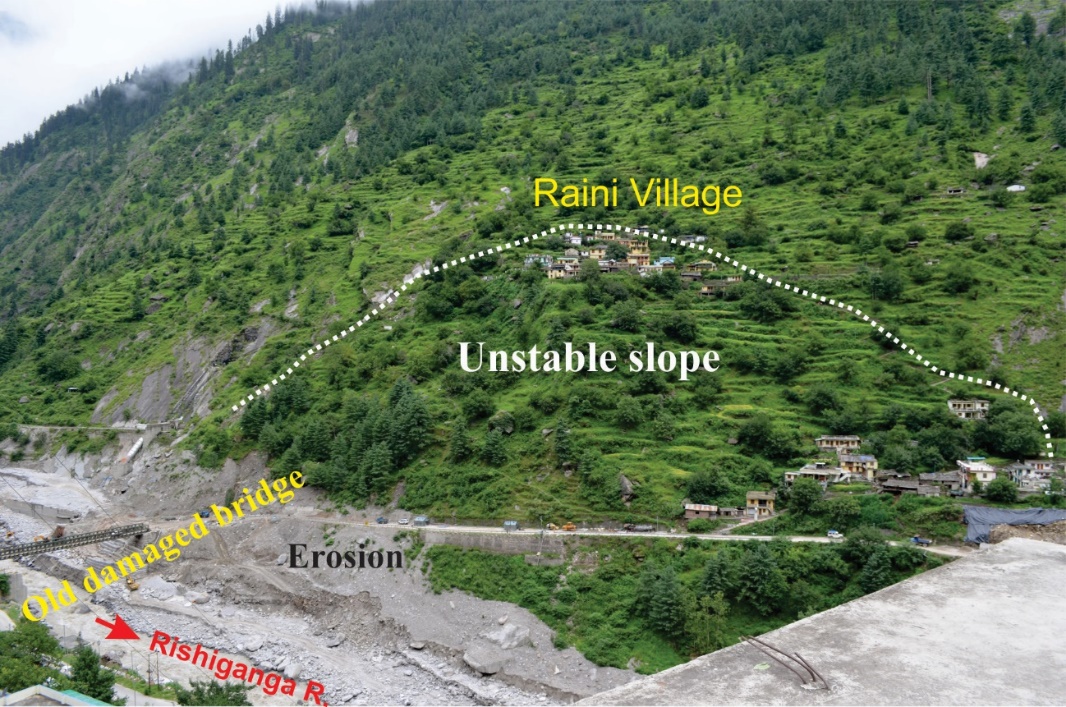
**

**Figure S2.** Field photograph (July 2021) shows devastating sites at Raini village after the 7^th^ February 2021 disaster in Chamoli, Uttarakhand.


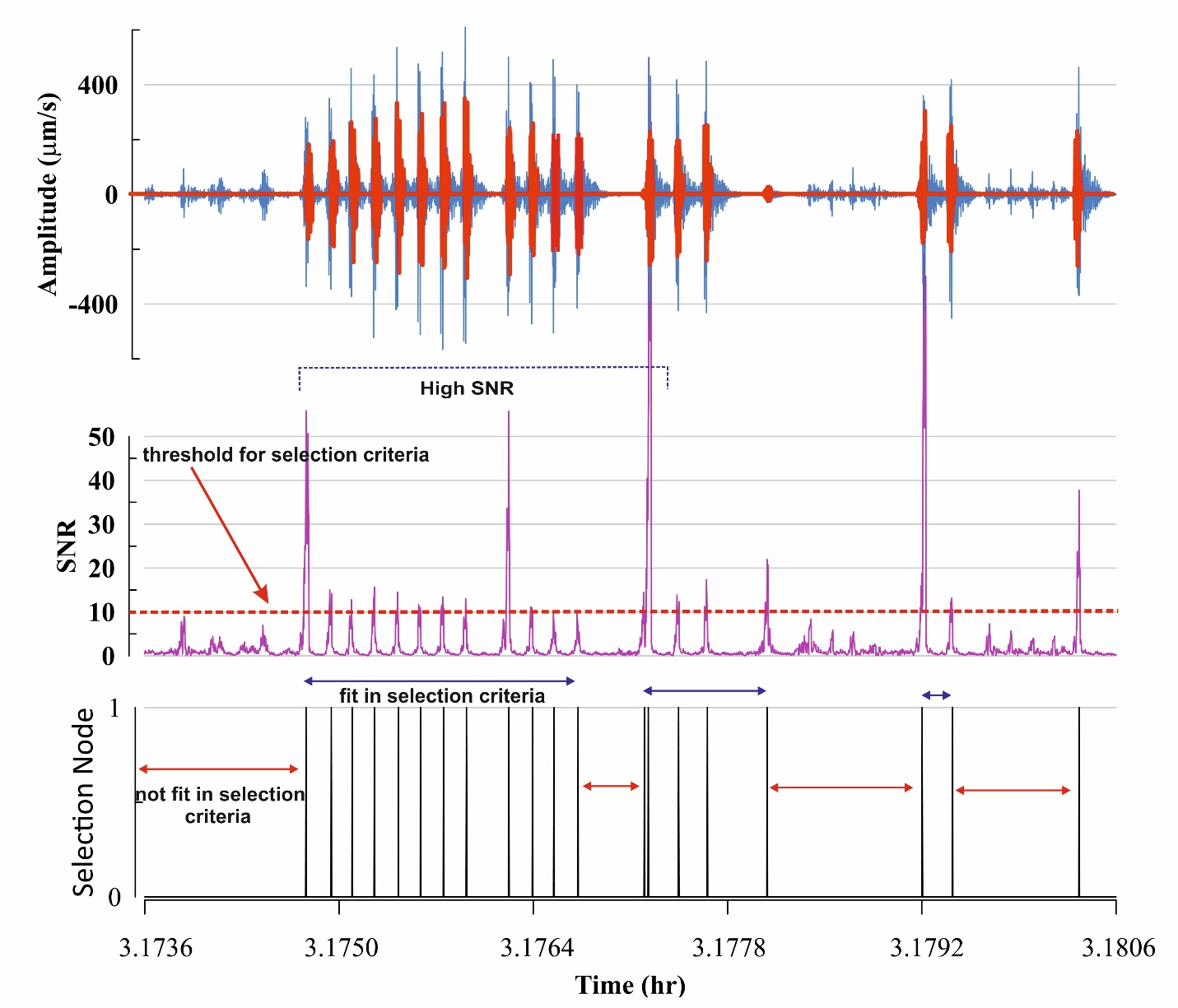


**Figure S3.** Selection criteria of precursory tremors based on SNR. A selection node detects those signals which follow the threshold of SNR>10 with high amplitude waveform. While other signals/tremors are considered as ‘not fit in selection criteria’.


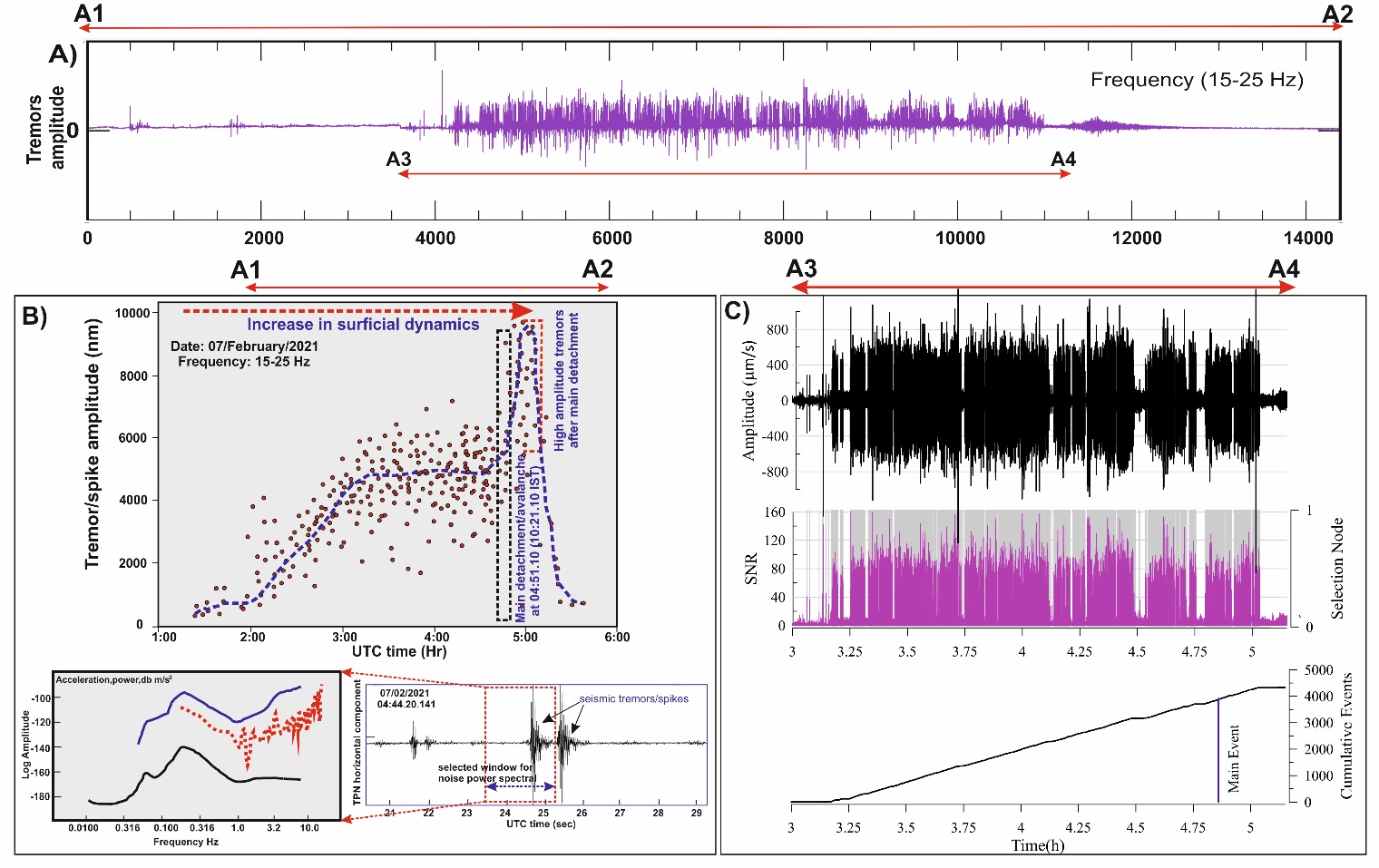


**Figure S4. (A)** The long hours seismic window showing cluster of events that are dominant with high amplitude and high SNR after ~03:00 Hr on 07/02/2021. **(B)** Showing the abrupt elevation of seismic amplitude and increase in seismic tremors. A standard random tremor is selected with noise power spectral density plot to show the selection criteria for tremors to best fit in Peterson model of noise. **(C)** A cumulative number of detected precursory events showing the huge number of detected tremors with high SNR prior to the main failure. The selection criteria is based on SNR threshold and amplitude variation.

**
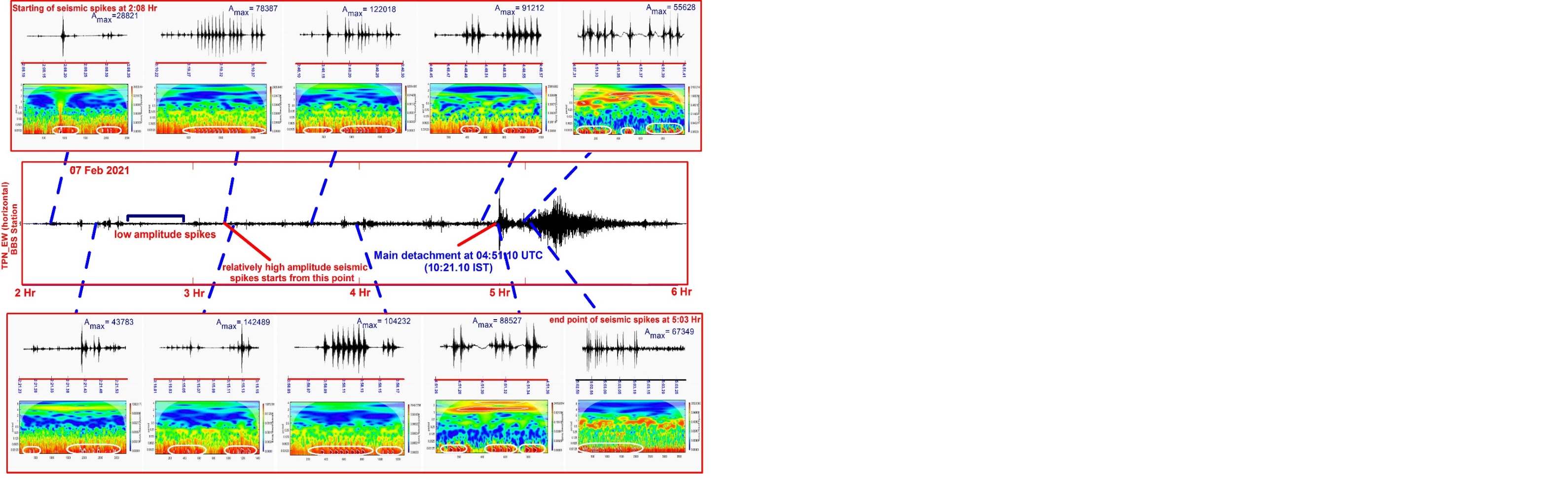
**

**Figure S5.** Frequency-time representation. Seismic precursory and continuous spikes were recorded in the TPN station before the main release. Frequency-time-spectral density plot (wavelet analysis) and seismic window (A_max_ in m/s^2^) show the continuous high amplitude seismic spikes (in counts) started at 2:08 Hr. UTC (7:38 AM IST: 7^th^ Feb. 2021) and ends at 5:03 Hr. UTC (10:33 AM IST: 7^th^ Feb. 2021). The white elliptical circle (bottom of the color plot) denotes the continuation of high frequency (>32 Hz) impact that was dominant throughout the seismic waveform window.


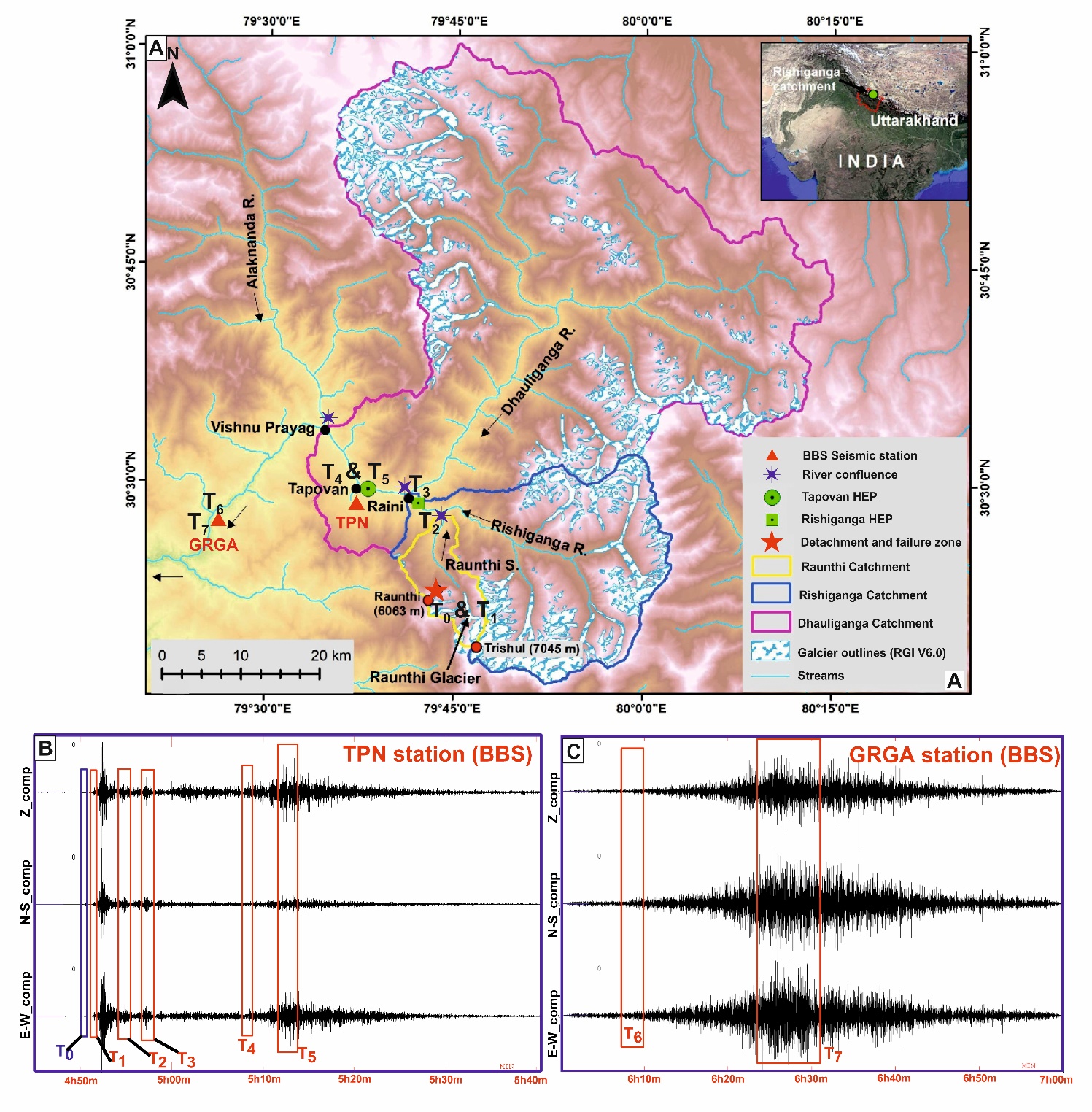


**Figure S6. (A)** Showing the locations of debris flow hitting impacts and material failure from the source zone in Rishiganga-Dhauliganga and Alaknanda valley. **(B & C)**The plot depicts the series of seismic signals (>15 Hz) recorded in two BBS stations (TPN and GRGA), close to the avalanche release zone and subsequent impacts. (T_1_-T_7_) chronological hitting impacts (change in momentum) recorded as changes in seismic spikes. Map was generated using ArcGIS software licensed version 10.5 (<https://www.arcgis.com/index.html>)

**
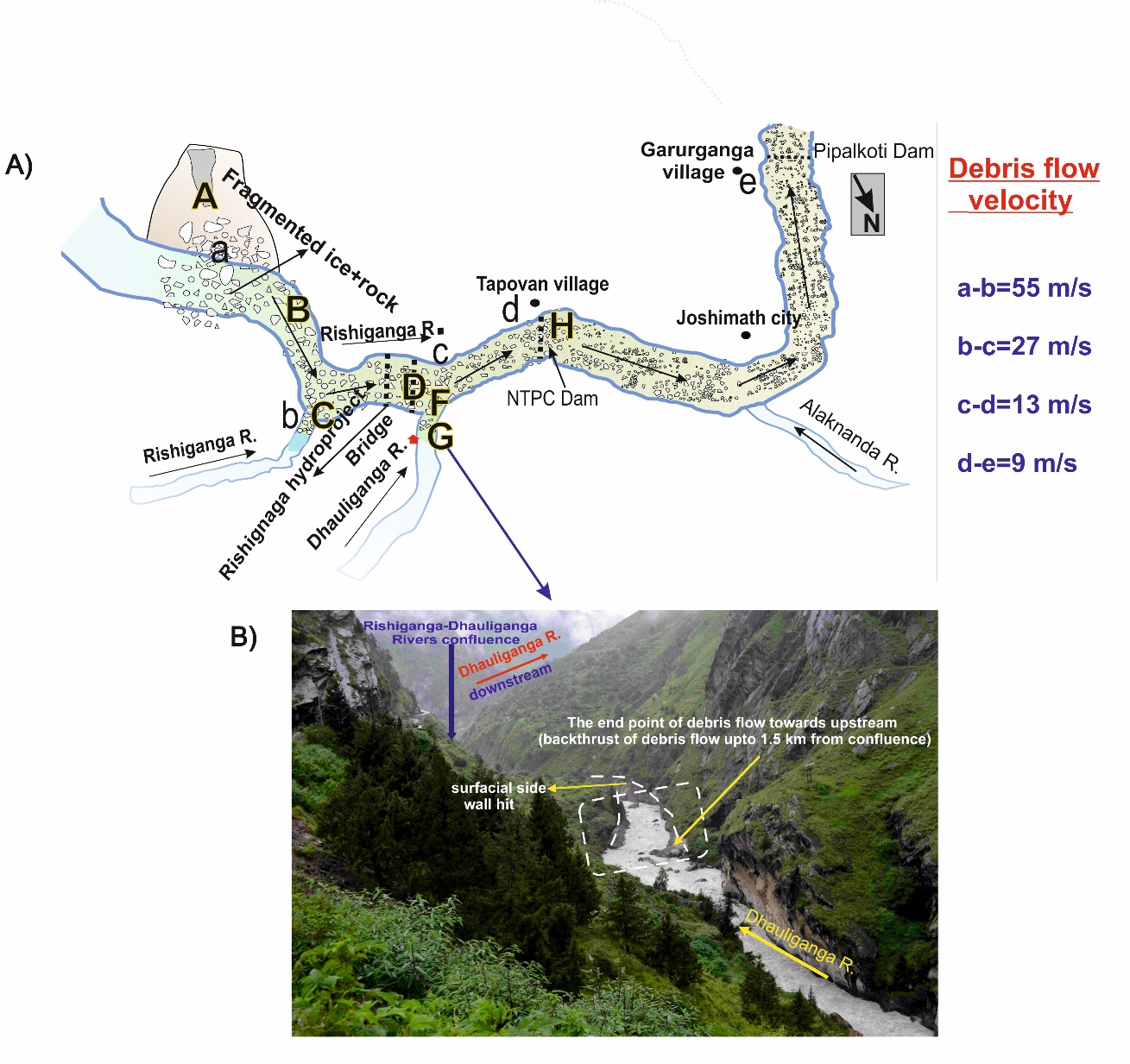
**

**Figure S7. (A)** Schematic diagram represents the successive path of debris flow with change in flow velocities. The field photographs is shown in figure 6 for the mentioned sequence (A-H) in schematic diagram. **(B)** Field photograph of Dhauliganga valley. The dotted white square depicts the affected valley and end point of debris flow in the upstream, from the confluence of Dhauliganga-Rishiganga River on 7 February 2021. The flow dynamicity affects the upstream of Dhauliganga River up to 1.5 km from the confluence point.

**References**

1. Havskov, J. & Ottemøller, L. SEISAN earthquake analysis software. *Seismol. Res. Lett.* **70**, 532–534 (1999).
2. Meena, S.R., Bhuyan, K., Chauhan, A. & Singh, R.P. Snow covered with dust after Chamoli rockslide: inference based on high-resolution satellite data. Rem. *Sens. Lett.* **12**(7), 704-714 (2021).
3. Gibbons, S.J. & Ringdal, F. The detection of low magnitude seismic events using array-based waveform correlation. *Geophys. J. Int.* **165**(1), 149– 166 (2006).
4. RoeschA, A., Schmidbauer, H. & Roesch, M.A. Package ‘WaveletComp’, <http://www.stats.bris.ac.uk/R/web/packages/WaveletComp/WaveletComp.pdf> (2014).
5. Torrence, C. & Compo, G. P. A practical guide to wavelet analysis. *Bull of the Am Meteor. Soc.* **79**, 6178 (1998).
6. Knapp, C. & Carter, G. The generalized correlation method for estimation of time delay IEEE Trans. *Acoust. Speech Signal Process.* **24** (4), 320-327 (1976).
7. Souden, M., Benesty, J. & Affes, S. Broadband source localization from an eigen analysis perspective IEEE Trans. *Audio Speech Lang. Process.*  **18**(6), 1575-1587 (2010).
